# Evaluation of Real-Time PCR Coupled With Multiplex Probe Melting Curve Analysis for Pathogen Detection in Patients With Suspected Bloodstream Infections

**DOI:** 10.3389/fcimb.2019.00361

**Published:** 2019-10-22

**Authors:** Yao Xiao, Xu Shen, Qi-Feng Zhao, Yi-Hui Yao, Tian-Ci Yang, Jian-Jun Niu

**Affiliations:** ^1^Center of Clinical Laboratory, Zhongshan Hospital, School of Medicine, Xiamen University, Xiamen, China; ^2^Xiamen Hospital of Traditional Chinese Medicine, Xiamen, China; ^3^Shaoxing Center for Disease Control and Prevention, Shaoxing, China; ^4^Institute of Infectious Disease, School of Medicine, Xiamen University, Xiamen, China

**Keywords:** PCR-MCA, bloodstream infections, pathogen detection, diagnostic performance, blood culture

## Abstract

**Background:** This study aimed to evaluate real-time polymerase chain reaction coupled with multiplex probe melting curve analysis (PCR-MCA) for pathogen detection in patients with suspected bloodstream infections (BSIs).

**Methods:** A PCR-MCA assay was developed for simultaneous identification of 28 kinds of the most common pathogens and two resistance genes within a few hours. The diagnostic performance of the PCR-MCA assay was determined and compared to the results of blood culture.

**Results:** A total of 2,844 consecutive new episodes of suspected BSIs in 2,763 patients were included in this study. There were 269 episodes of pathogens identified by blood culture. For all the pathogens tested, the PCR-MCA assay exhibited a sensitivity of 88.8% (239/269), specificity of 100% (2,575/2,575), and agreement of 98.9% (2,814/2,844). For the pathogens on the PCR-MCA list, the PCR-MCA results had a sensitivity of 99.2% (239/241), specificity of 100% (2,575/2,575), and agreement of 99.9% (2,814/2,816) compared with the results of blood culture. For seven samples with multiple pathogens identified simultaneously during one blood culture investigation, the PCR-MCA assay verified the results of the blood culture, with an agreement rate of 100% for each.

**Conclusion:** The PCR-MCA assay could discover 88.8% of the pathogens in clinical practice, showing excellent diagnostic performance vs. that of blood culture for pathogen detection in patients with suspected BSIs, and would contribute to rapid diagnosis and correct antibiotic administration.

## Introduction

Sepsis could be called a severe complication of bloodstream infections (BSIs). The reported incidence of sepsis is increasing, and conservative estimates indicate that sepsis is a leading cause of mortality and critical illness worldwide (Singer et al., 2016). Early recognition of sepsis and accurate administration of antibiotics are important for improving outcomes. According to international guidelines (Singer et al., 2016), empirical antibiotic treatment should be given within 1 h once sepsis is suspected, and broad-spectrum antibiotics are often recommended. However, as early empirical treatment may be ineffective more often than in the past because of the worldwide rise and broadening of bacterial multidrug resistance (Gustinetti and Mikulska, 2016), precise diagnosis and antibiotic treatment targeting the causative pathogen of sepsis are urgently need.

Blood cultures, aiming to detect viable microorganisms in blood, are still considered as the “gold standard” for the microbiological diagnosis of BSIs. Suspected BSIs can be identified if the culture is obtained first and the antibiotic is concomitantly administered within 72 h, whereas if the antibiotic is first and the culture is acquired within 24 h (Singer et al., 2016). However, when antibiotics are administered before the culture is obtained, sensitivity is greatly reduced, and slow-growing, fastidious microorganisms are difficult to detect by blood culture (Opota et al., 2015). Studies have shown that only approximately half of the patients experiencing sepsis and septic shock have positive blood cultures (Bochud et al., 2001). Furthermore, ~24–72 h is required to identify the pathogen and obtain information on antibiotic sensitivity (Peters et al., 2004). As a consequence, empirical antibiotic therapy is more commonly guided by clinical response than by the result of a blood culture.

To achieve faster and more accurate diagnosis of sepsis, rapid molecular diagnostic tests have been developed for BSI diagnosis. The U.S. Food and Drug Administration has cleared several multiplex molecular assays that can detect a wide range of microorganisms concurrently with specific resistance genes directly in blood samples, and some of these assays have been made commercially available (Rello et al., 2018). One of the most developed technologies is the multiplex polymerase chain reaction (PCR) assay, which can detect the DNA of as many as 20 pathogen species directly in blood samples within hours (Galiana et al., 2017; Makristathis et al., 2018), even more kinds of microorganisms concurrently with several resistance genes in positive blood cultures (Ramanan et al., 2018). Recently, multiplex probe melting curve analysis (MCA) based on real-time PCR assays has also been applied in pathogen identification (Jiang et al., 2017), allowing simultaneous identification of ten bacterial pathogens in gastroenteritis and wound infections via a single reaction. On this basis, we developed a real-time PCR coupled with multiplex probe MCA (PCR-MCA) assay for simultaneous identification of 28 kinds of the most common pathogens and two resistance genes in BSIs. After the multiplex ligation reaction, linear-after-the-exponential PCR was carried out with a real-time PCR instrument by using the fluorophore and melting temperature (*Tm*) value as dual indicators, and MCA yielded a unique *Tm* code by which a certain pathogenic bacterium could be identified (Liao et al., 2013). In this study, we aimed to evaluate the clinical performance of this assay compared with that of blood culture for pathogen detection in patients with suspected BSIs.

## Materials and Methods

### Study Population and Ethics Statement

This study was conducted at Zhongshan Hospital, School of Medicine, Xiamen University, from October 2016 to June 2017. The inclusion criteria for the study population were as follows: clinical suspicion of the development of life-threatening organ dysfunction caused by a BSI identified by a treating clinician and an urgent need for blood cultures. For clinical operationalization, organ dysfunction can be represented by an increase of 2 points or more in the sepsis-related organ failure assessment score (Singer et al., 2016). Patients who had been administered with antibiotics (such as some postoperative or transferred patients) were excluded from the study. In this study, the observation unit was an episode of suspected BSI, the results were evaluated as follows: once a bacterial or fungal pathogen was isolated from at least one blood culture, a positive BSI episode was recorded, and the consecutive duplicate results were merged; a BSI episode was considered new if it was detected at least 14 days from the previous infection with positive blood culture or if a different microorganism was isolated from a new blood sample (Quiles et al., 2019); consecutive repeated negative blood culture results from one patient during one hospitalization was recorded as a negative BSI episode. A total of 2,763 patients with 2,844 consecutive new episodes of suspected BSIs were recruited. This study was approved by the Institutional Ethics Committee of Zhongshan Hospital, School of Medicine, Xiamen University, and was in compliance with national legislation and the Declaration of Helsinki guidelines. Written informed consent was obtained from the patient or, from next of kin or an independent patient advocate if the patient lacked this capacity.

### Laboratory Procedures

Blood samples were obtained from eligible patients as follows. First, two blood samples were collected simultaneously from peripheral venepuncture (8–10 mL for adults and 1 mL for children) and inoculated into paired culture bottles (Plus Aerobic, Plus Anaerobic; BD BACTEC^TM^). Blood culture entered the standard clinical pathway, and the results were returned directly to the clinical service at each center. Second, research blood samples (5 mL for adults and 1 mL for children) were obtained from the same venepuncture sites at the same time and were placed into sterile EDTA tubes for PCR-MCA analysis of pathogen DNA in whole blood. The research blood samples were transferred to a single molecular laboratory and kept for no more than 72 h at 4°C prior to PCR analysis.

### Blood Culture

Blood culture bottles were incubated for 5 days in an automated blood culture instrument (BD Bactec^TM^ FX). When bacterial growth was noted, the positive culture sample was inoculated into blood agar, MacConkey agar and chocolate agar and then cultured overnight in a 37°C electro-heating standing-temperature cultivator. A single colony of fresh overnight culture was smeared directly onto a ground-steel matrix-assisted laser desorption/ionization (MALDI) target plate, covered with 1.5 μL of saturated α-cyano-4-hydroxycinnamic acid matrix in 50% acetonitrile-2.5% trifluoroacetic acid and allowed to dry. The plate was inserted into the source of a Bruker Microflex LT/SH MALDI-TOF-MS instrument (Bruker MALDI Biotyper) for identification. The blood culture was considered negative if no bacterial growth was detected within 5 days and was not evaluated by the Bruker MALDI Biotyper.

### DNA Preparation

The blood was cultured with a pipette at 1 mL in an EP tube and centrifuged at 13,000 × g for 2 min to precipitate the bacteria. After the supernatant was removed by pipetting, 100 μL of 1 × TE buffer (10 mM Tris base, 1 mM EDTA, pH 8.5) was added to the bacterial precipitate. The EP tube was heated at 99°C for 10 min and centrifuged at 13,000 × g for 10 min. The supernatant was removed into the new EP tube and stored in a −20°C freezer for PCR-MCA.

### PCR-MCA

In this study, tagged primers were used, which were different from universal primers in that they contained a common tag sequence at the 5′ ends of specific primers. The primer sequences (Sangon Biotech (Shanghai) Co., Ltd.) are shown in Table 1; these primers target 12 kinds of gram-positive bacteria, 11 kinds of gram-negative bacteria, five kinds of fungi, and two resistance genes, namely, a vancomycin resistance-encoding gene for *Enterococcus* species (*vanA*) and a beta-lactam resistance-encoding gene for *Staphylococcus* species (*mecA*). The probes in this study were composed of media probes and universal probes. The secondary structures of the probes were predicted using *mfold Web Server* online analysis software (http://mfold.rna.albany.edu/?q=mfold/DNA-Folding-Form). The fluorophores FAM, ROX, CY5, and HEX were marked with a 5′ universal probe, and the quenchers BHQ1 and BHQ2 were marked with a 3′ probe. The probe sequences have not been shown here as the patent is pending. The *Tm* values of the probes were predicted using Tm Utility v1.3 software. The homology of the designed primers and probes was tested using BLAST software (https://blast.ncbi.nlm.nih.gov/Blast.cgi).

**Table 1:** Primer sequences for the pathogens in the PCR-MCA system.

**Pathogens**		**Primer sequences**	Tm^*^ (^**°**^C)
**Gram negative**			
	Tag	GCAACGGGTCAGGTAGCCATCTC	
*Escherichia coli*	Forward	Tag-ATCGATCCATATTTACCATATCAATACTTG	62.09
	Reverse	Tag-ATCCAGTATGTTCAAATCCTAAGTTACTC	
*Klebsiella pneumoniae*	Forward	Tag-GGTAAAATGAGTTTGCCGC	61.21
	Reverse	Tag-ACCAGGATATTCAGCAGCAT	
*Acinetobacter baumannii*	Forward	Tag-ATGGTGAAATTCGTGGTAAAGA	69.60
	Reverse	Tag-GGCGAGATGGTTTACTCACTA	
*Pseudomonas aeruginosa*	Forward	Tag-CTTGAGGTAGGGACCGAG	81.80
	Reverse	Tag-CGACGAGCTGTACATGGT	
*Enterobacter cloacae*	Forward	Tag-TCAGGTAAGTTATTCATCATCATCTTAT	80.61
	Reverse	Tag-TAAACGCTGGTTTTTTCATAAT	
*Klebsiella oxytoca*	Forward	Tag-GATCGTCCGAACCCAGCAG	76.06
	Reverse	Tag-CGGCGGCGAATTCAAAAC	
*Enterobacter aerogenes*	Forward	Tag-GCGGCAAAGTCCATATATAAACT	67.23
	Reverse	Tag-AGAAAATCATAGGGATAGCGTAGT	
*Haemophilus parainfluenzae*	Forward	Tag-CTTTGATGACGAGTGGCGGA	–
	Reverse	Tag-TCCCACTTGGGCTCATCCTA	
*Citrobacter*	Forward	Tag-CGTGTAACTCTTATCAGAATATCAGC	64.30
	Reverse	Tag-AAAAACGAGATTATACCGTAACCCT	
*Serratia marcescens*	Forward	Tag-TTCCTGTTCCTGGAAGAC	67.26
	Reverse	Tag-GTTCTTGTCGTTTTCCACC	
*Proteus mirabilis*	Forward	Tag-AGTAAAGAGAACACAGATTGTAA	80.58
	Reverse	Tag-TCACAGTCACCACTAATCTCAC	
**Gram positive**			
	Tag	GCAACGGGTCAGGTAGCCATCTC	
*Staphylococcus aureus*	Forward	Tag-ATCGATCCATATTTACCATATCAATACTTG	79.14
	Reverse	Tag-ATCCAGTATGTTCAAATCCTAAGTTACTC	
*Staphylococcus epidermidis*	Forward	Tag-CCCCATCTTTTTAAGTGTTTAGGAAT	79.72
	Reverse	Tag-ATCAGTAATGGGAAATAAATCCATATTGA	
*Streptococcus pneumoniae*	Forward	Tag-GGTATTGTTGGTTATGTACTTGTGT	75.51
	Reverse	Tag-ATTTCTCTTTATCTTTTGCTTTGG	
*Staphylococcus capitis*	Forward	Tag-GCTGGACTAAAAAGTATAAGAGTATACTGATTA	74.87
	Reverse	Tag-ATCCAAGTATTTTTCTAATCGCGTATCT	
*Enterococcus faecalis*	Forward	Tag-GACGAAAGTCTGACCGAGCA	–
	Reverse	Tag-TGCCACCTACGTATTACCGC	
*Enterococcus faecium*	Forward	Tag-TTGGAATGAAGTTGGCGGGA	–
	Reverse	Tag-GCTGCCGAACAGTTCCTGAT	
*Streptococcus dysgalactiae*	Forward	Tag-GAAATGGAACACGTTAGGGTCG	–
	Reverse	Tag-TCTTAACTAGAAAAACTCTTGATTATTC	
*Streptococcus anginosus*	Forward	Tag-GTTTTGCAGAAGCGATTGTC	–
	Reverse	Tag-TGAGAGCACGGTTTGAGTCG	
*Staphylococcus haemolyticus*	Forward	Tag-TGAATCTGGTGGCGACATTAC	75.87
	Reverse	Tag-AGGAGCTACTGAATCCCAAGTA	
*Streptococcus mutans*	Forward	Tag-CCTACGGGAGGCAGCAGTAG	–
	Reverse	Tag-CAACAGAGCTTTACGATCCGAAA	
*Streptococcus pyogenes*	Forward	Tag-CACCTTACTAACTGAACAATCTT	74.52
	Reverse	Tag-GTGTAGTGGAGGTAGAAGGTT	
*Listeria*	Forward	Tag-GCCTTTTTCATTGGTAGCATC	63.82
	Reverse	Tag-GCAAGGTAGAGGATATAGAGAAG	
**Fungus**			
	Tag	GCAACGGGTCAGGTAGCCATCTC	
*Candida albicans*	Forward	Tag-GCCATCTAGGAGTAAACCACAAC	59.30
	Reverse	Tag-CAGCCAGGGTAGCTCATG	
*Candida glabrata*	Forward	Tag-ATCTAAGGCTAAATACTGGCGA	79.50
	Reverse	Tag-CAATACCCTTCCCTTTCAACA	
*Candida parapsilosis*	Forward	Tag-GTCGTGGTCTTCAGTAACTAACA	84.66
	Reverse	Tag-AAGATTCAGCCAACCACAAGATG	
*Candida krusei*	Forward	Tag-CTCGGAATGTATCACCTCT	82.08
	Reverse	Tag-TTTACGACCATTACGCCAG	
*Aspergillus fumigatus*	Forward	Tag-ATCAGTATGTGGGACACGAG	69.45
	Reverse	Tag-CGAGTTGGTAAAACCTAATACAGT	
**Resistance gene**			
	Tag	GCAACGGGTCAGGTAGCCATCTC	
*mecA*	Forward	Tag-GTGATTATCCATTTTATAATGCTCA	85.19
	Reverse	Tag-GCCATTATTTTCTAATGCGCTAT	
*vanA*	Forward	Tag-GGTGTATGGAAAATGTGCGAA	74.44
	Reverse	Tag-GGCGAGAGTACAGCTGAATAG	
**Internal control**			
	Forward	GCCATCTCTCTCTATTAGTTCGT	70.91
	Reverse	GCCATCTCTCTCTATTAGTTCGT	

*PCR-MCA, real-time polymerase chain reaction coupled with multiplex probe melting curve analysis; vanA, vancomycin resistance-encoding gene for Enterococcus species; mecA, beta-lactam resistance-encoding gene for Staphylococcus species*.

* *The Tm values for Haemophilus parainfluenzae, Enterococcus faecalis, Enterococcus faecium, Streptococcus dysgalactiae, Streptococcus anginosus, and Streptococcus mutans are not shown because the patent is pending*.

When the uniplex PCR assay was established, three pairs of primers were designed for each testing object first, and primers were screened by pairwise combination to select the optimal primer combination. Second, the PCR components were optimized. The final 25-μL uniplex PCR assay included 5.0 mM MgCl_2_, 1× buffer (67 mM Tris-HCl, 16.6 mM (NH_4_)_2_SO_4_, 6.7 μM EDTA, and 0.085 mg/mL BSA), 0.2 mM dNTPs, 2.0 U of Taq01 DNA polymerase, 0.01 U of UNG enzyme, 0.05 μM forward/reverse primers, 0.4 μM universal primers, 0.3 μM media probes, 0.08 μM universal probes, and 5 μL of template DNA. After the PCR procedure was optimized, the procedure used for the uniplex PCR assay for BSIs was as follows: amplification stage: 50°C for 2 min, predenaturation at 95°C for 5 min, 95°C for 20 s and 60°C for 1 min (for a total of 50 cycles), and final extension at 35°C for 25 min; melting stage: denaturation at 95°C for 2 min and heat preservation at 45°C for 2 min, followed by probe melting curve analysis via a temperature ramp from 45 to 95°C with a heating rate of 0.04°C/s. Fluorescence signals in the corresponding detection channels were collected during the whole melting process.

On the basis of the uniplex PCR assay for BSIs, all primers and probes were combined to establish the multiplex PCR assay. The PCR components were optimized, and the final 25-μL multiplex PCR assay included 5.0 mM MgCl_2_, 1× buffer, 0.25 mM dNTPs, 2.5 U of Taq01 DNA polymerase, 0.01 U of UNG enzyme, 0.7 μM universal primers, 7 universal probes at 0.1 μM each, and 5 μL of template DNA. After the PCR procedure was optimized, the procedure used for the multiplex PCR assay for BSIs was the same as that for the uniplex PCR assay, and the fluorescence signals in the corresponding detection channels were collected during the whole melting process. The uniplex and multiplex experiments were carried out on an SLAN 96S real-time fluorescence PCR instrument (Shanghai Hongshi Medical Technology Co., Ltd.). Discordant results between the PCR-MCA assay and blood culture were further verified using a uniplex PCR assay.

### Analytical Evaluation

To evaluate analytical sensitivity, first, plasmids of 28 kinds of pathogens and two resistance genes were constructed, and serial dilutions of the DNA were prepared from 10^4^ copies/μL to 10^0^ copies/μL. Second, suspensions of reference strains of the 28 kinds of pathogens were prepared with serial 10-fold dilutions in 0.9% NaCl. Each dilution, the concentration was determined by plate counts (CFU/mL), was subjected to DNA extraction. The limit of detection (LOD) was defined as the lowest concentration that yielded no more than one negative result in 20 replicates (i.e., positive rate, ≥95%).

To evaluate analytical specificity, each target pathogen or resistance gene in the detection system was used as a template for the PCR. In addition, a blind test of the bacterial samples, including the 28 targeted pathogens and non-targeted pathogens, was performed.

The reproducibility was evaluated using the constructed plasmids for each pathogen or resistant gene as templates (concentration 10^3^ copies/μL). The experiments were carried out three times on the same instrument, with three duplicates of each template for each experiment. The Tm values of the melting peaks of each test object in different experiments and parallel tubes were analyzed by calculating the standard deviations (SDs) and coefficient of variation (CV).

### Statistical Analysis

The results of blood culture for identification of pathogens were used as the gold standard in this study. The diagnostic performance of the PCR-MCA assay was determined and compared to the results of blood culture, using sensitivity, specificity, positive predictive value, negative predictive value (NPV), agreement rate and Kappa value, according to the Yerushalmy model (Niu et al., 2015). The sensitivity was calculated as the number of episodes in which both blood culture and PCR-MCA were positive divided by the sum of these episodes and the number of episodes in which blood culture was positive but PCR-MCA were negative. The specificity was calculated as the number of episodes in which both blood culture and PCR-MCA were negative divided by the sum of these episodes and the number of episodes in which blood culture was negative but PCR-MCA were positive. The statistical analyses were performed using SPSS 19.0 for Windows (SPSS Inc., Chicago, Illinois, USA).

## Results

### Characteristics of the Study Participants

A total of 2,763 patients with 2,844 consecutive new episodes of suspected BSIs were recruited in this study. The median age of patient episodes was 44 years (interquartile range, 1–67 years), including 1,658 male episodes (58.3%) and 1,186 female episodes (41.7%). The main proportion of participants came from the pediatrics department (33.5%), followed by the pulmonology department (16.7%), general surgery department (10.2%), hematology department (6.6%), gastroenterology department (6.2%), nephrology department (5.3%), etc (Table 2). The prevalence of BSIs identified by blood culture was 26.5% (148/558) in surgery, 7.5% (93/1,248) in internal medicine, 11.9% (10/84) in obstetrics and gynecology, and 1.9% (18/954) in pediatrics.

**Table 2:** Characteristics of the study participants.

		**Specialties**											
	**Entire cohort**	**Surgery**				**Internal medicine**						**Obstetrics and gynecology**	**Pediatrics**
		**General surgery**	**Urology**	**Orthopedic surgery**	**Neurosurgery**	**Pulmonology**	**Hematology**	**Gastroenterology**	**Nephrology**	**Neurology**	Other internal medicine^*^		
Patient episodes, *n* (%)	2,844	289 (10.2)	114 (4.0)	63 (2.2)	92 (3.2)	474 (16.7)	188 (6.6)	176 (6.2)	151 (5.3)	128 (4.5)	131 (4.6)	84 (3.0)	954 (33.5)
Male episodes, *n* (%)	1,658 (58.3)	179 (61.9)	68 (59.6)	46 (73.0)	59 (64.1)	330 (69.6)	111 (59.0)	118 (67.0)	73 (48.3)	82 (64.1)	60 (45.8)	0 (0.0)	532 (55.8)
Age, M (IQR) (years)	44 (1–67)	60 (51–70)	60 (49–69)	49 (32–63)	52 (39–62)	66 (52–80)	57 (32–71)	63 (47–76)	61 (48–73)	68 (54–78)	58 (46–70)	32 (28–39)	0 (0–2)
Blood stream infection episodes, *n* (%)	269 (9.5)	104 (36.0)	21 (18.4)	13 (20.6)	10 (10.9)	28 (5.9)	13 (6.9)	8 (4.5)	17 (11.3)	12 (9.4)	15 (11.5)	10 (11.9)	18 (1.9)

*M, median; IQR, interquartile range*.

* *Other internal medicine included rheumatology (64 patient episodes), endocrinology (53 patient episodes), and cardiology (14 patient episodes)*.

### Performance of the PCR-MCA Assay for Identification of Pathogens Compared to That of Blood Culture

The analytical sensitivity, specificity and reproducibility of the PCR-MCA assay were evaluated. The results showed that the analytical sensitivity reached 50 to 5 copies per reaction. The 28 targeted pathogens gave reproducible readouts in 20 replicates when the concentration was equal to or higher than 300 CFU/mL; therefore, the PCR-MCA assay LOD was determined to be 300 CFU/mL (data not shown). The analytical specificity results showed that the PCR-MCA assay could correctly detect all 28 targeted pathogens and exhibited no cross-reactivity with non-targeted pathogens. The relevant melting signal peak of each pathogen or resistance gene was achieved, and no interference existed among the signals. The reproducibility results showed that the SDs of the *Tm* values were no more than 1°C and that the CVs were no more than 1%, indicating a high reproducibility of this assay.

Among the 2,844 episodes of suspected BSIs, 269 were identified for the presence of pathogens with the results of blood culture. The most common gram-negative pathogen was *Escherichia coli* (*n* = 95, 35.3%), followed by *Klebsiella pneumoniae* (*n* = 40, 14.9%), *Acinetobacter baumannii* (*n* = 9, 3.3%), *Pseudomonas aeruginosa* (*n* = 9, 3.3%), *Enterobacter cloacae* (*n* = 5, 1.9%), etc. The most common gram-positive pathogen was *Staphylococcus aureus* (*n* = 22, 8.2%), followed by *Staphylococcus epidermidis* (*n* = 16, 5.9%), *Streptococcus pneumoniae* (*n* = 9, 3.3%), *Staphylococcus capitis* (*n* = 7, 2.6%), *Enterococcus faecium* (*n* = 6, 2.2%), *Enterococcus faecalis* (*n* = 6, 2.2%), etc. The most common fungus was *Candida albicans* (*n* = 8, 3.0%). Among the 2,844 episodes of suspected BSIs, 24 episodes were identified with *Staphylococcus* species (including 11 with *S. epidermidis*, five with *S. capitis*, four with *S. aureus*, two with *S. haemolyticus*, one with *S. hominis*, and one with *S. lugdunensis*) resistant to all beta-lactam antibiotics in phenotypic susceptibility tests. However, the *mecA* gene was not detected in any of these species using the PCR-MCA assay. In addition, no vancomycin-resistant *Enterococcus* species were identified in phenotypic susceptibility tests, consistent with the PCR-MCA result showing that the *vanA* gene was not detected. The performance of the PCR-MCA assay compared to that of blood culture for identification of each pathogen is shown in Table 3. For all the pathogens tested, the PCR-MCA assay discovered 88.8% (239/269) of the pathogens discovered by blood culture, with a sensitivity of 88.8%, specificity of 100% (2,575/2,575), and agreement of 98.9% (2,814/2,844). Among the 269 episodes of pathogens identified by blood culture, 241 were identified with a pathogen on the PCR-MCA list, while 28 were not. For the pathogens on the PCR-MCA list, the PCR-MCA assay had a sensitivity of 99.2% (239/241), specificity of 100% (2,575/2,575), and agreement of 99.9% (2,814/2,816) compared with the results of blood culture.

**Table 3:** Performance of the PCR-MCA assay for identification of pathogens compared to the results of blood culture.

**Pathogens**	**Blood culture/PCR-MCA**				**Sensitivity (%)**	**Specificity (%)**	**PPV (%)**	**NPV (%)**	**Agreement rate (%)**	**Kappa value**
	****+/+^a^****	****+/−^b^****	****−/+^c^****	****−/−^d^****						
**Pathogens on the PCR-MCA List**										
**Gram negative**										
*E. coli*	95	0	1	2,748	100.00	99.96	99.00	100.00	99.96	0.995
*K. pneumoniae*	38	2	1	2,803	95.00	99.96	97.40	99.93	99.89	0.961
*A. baumannii*	9	0	2	2,833	100.00	99.93	81.80	100.00	99.93	0.900
*P. aeruginosa*	9	0	0	2,835	100.00	100.00	100.00	100.00	100.00	1.000
*E. cloacae*	5	0	0	2,839	100.00	100.00	100.00	100.00	100.00	1.000
*K. oxytoca*	2	0	0	2,842	100.00	100.00	100.00	100.00	100.00	1.000
*E. aerogenes*	2	0	0	2,842	100.00	100.00	100.00	100.00	100.00	1.000
*H. parainfluenzae*	1	0	0	2,843	100.00	100.00	100.00	100.00	100.00	1.000
*Citrobacter*	1	0	0	2,843	100.00	100.00	100.00	100.00	100.00	1.000
*S. marcescens*	0	0	0	2,844	n.a.	100.00	n.a.	100.00	100.00	n.a.
*P. mirabilis*	0	0	0	2,844	n.a.	100.00	n.a.	100.00	100.00	n.a.
**Gram positive**										
*S. aureus*	22	0	0	2,822	100.00	100.00	100.00	100.00	100.00	1.000
*S. epidermidis*	16	0	0	2,828	100.00	100.00	100.00	100.00	100.00	1.000
*S. pneumoniae*	9	0	1	2,834	100.00	99.96	90.00	100.00	99.96	0.947
*S. capitis*	7	0	0	2,837	100.00	100.00	100.00	100.00	100.00	1.000
*E. faecium*	6	0	0	2,838	100.00	100.00	100.00	100.00	100.00	1.000
*E. faecalis*	6	0	1	2,837	100.00	99.96	85.70	100.00	99.96	0.923
*S. dysgalactiae*	3	0	0	2,841	100.00	100.00	100.00	100.00	100.00	1.000
*S. anginosus*	3	0	0	2,841	100.00	100.00	100.00	100.00	100.00	1.000
*S. haemolyticus*	2	0	0	2,842	100.00	100.00	100.00	100.00	100.00	1.000
*S. mutans*	1	0	0	2,843	100.00	100.00	100.00	100.00	100.00	1.000
*S. pyogenes*	0	0	0	2,844	n.a.	100.00	n.a.	100.00	100.00	n.a.
*L. monocytogenes*	0	0	0	2,844	n.a.	100.00	n.a.	100.00	100.00	n.a.
**Fungus**										
*C. albicans*	8	0	0	2,836	100.00	100.00	100.00	100.00	100.00	1.000
*C. glabrata*	1	0	0	2,843	100.00	100.00	100.00	100.00	100.00	1.000
*C. parapsilosis*	0	0	0	2,844	n.a.	100.00	n.a.	100.00	100.00	n.a.
*C. krusei*	0	0	0	2,844	n.a.	100.00	n.a.	100.00	100.00	n.a.
*A. fumigatus*	0	0	0	2,844	n.a.	100.00	n.a.	100.00	100.00	n.a.
**Pathogens not on the PCR-MCA List**										
*Streptococcus constellatus*	0	4	0	2,840	0.00	100.00	n.a.	99.86	99.86	n.a.
*Burkholderia cepacia*	0	3	0	2,841	0.00	100.00	n.a.	99.89	99.89	n.a.
*Bacteroides fragilis*	0	2	0	2,842	0.00	100.00	n.a.	99.93	99.93	n.a.
*Salmonella typhimurium*	0	2	0	2,842	0.00	100.00	n.a.	99.93	99.93	n.a.
*Staphylococcus hominis*	0	2	0	2,842	0.00	100.00	n.a.	99.93	99.93	n.a.
*Streptococcus mitis*	0	2	0	2,842	0.00	100.00	n.a.	99.93	99.93	n.a.
*Aeromonas hydrophila*	0	1	0	2,843	0.00	100.00	n.a.	99.96	99.96	n.a.
*Atopobium*	0	1	0	2,843	0.00	100.00	n.a.	99.96	99.96	n.a.
*Clostridium difficile*	0	1	0	2,843	0.00	100.00	n.a.	99.96	99.96	n.a.
*Corynebacterium striatum*	0	1	0	2,843	0.00	100.00	n.a.	99.96	99.96	n.a.
*Enterococcus avium*	0	1	0	2,843	0.00	100.00	n.a.	99.96	99.96	n.a.
*Enterococcus casselifavus*	0	1	0	2,843	0.00	100.00	n.a.	99.96	99.96	n.a.
*Kingella denitrificans*	0	1	0	2,843	0.00	100.00	n.a.	99.96	99.96	n.a.
*Micrococcus*	0	1	0	2,843	0.00	100.00	n.a.	99.96	99.96	n.a.
Non-fermenter	0	1	0	2,843	0.00	100.00	n.a.	99.96	99.96	n.a.
*Staphylococcus cohnii*	0	1	0	2,843	0.00	100.00	n.a.	99.96	99.96	n.a.
*Staphylococcus lugdunensis*	0	1	0	2,843	0.00	100.00	n.a.	99.96	99.96	n.a.
*Stenotrophomonas maltophilia*	0	1	0	2,843	0.00	100.00	n.a.	99.96	99.96	n.a.
*Streptococcus intermedius*	0	1	0	2,843	0.00	100.00	n.a.	99.96	99.96	n.a.

*PCR-MCA, real-time polymerase chain reaction coupled with multiplex probe melting curve analysis; PPV, positive predictive value; NPV, negative predictive value; n.a., not applicable*.

a *+/+ represents the number of episodes in which both blood culture and PCR-MCA were positive*.

b *+/− represents the number of episodes in which blood culture was positive but PCR-MCA was negative*.

c *−/+ represents the number of episodes in which blood culture was positive but PCR-MCA was negative*.

d *−/− represents the number of episodes in which both blood culture and PCR-MCA were negative*.

### Results of the Blood Culture and PCR-MCA Assay for the Identification of Multiple Infections

Among the 2,763 patients recruited, there were seven cases in whose blood samples two kinds of pathogens were identified simultaneously during one blood culture investigation. For these samples, the PCR-MCA assay verified the results of blood culture, with an agreement rate of 100% for each case. The results of blood culture and the PCR-MCA assay for the identification of multiple infections are shown in Table 4. In addition, there were eight subjects who underwent two or more sequential episodes of BSIs at different times during one hospitalization. In five cases (cases number 10, 82, 91, 94, and 133), the results of the PCR-MCA assay for identification of pathogens were consistent with those of blood culture in every infection episode. For case No. 9, the PCR-MCA assay verified the results of blood culture for the second and third infection episodes but showed negative results for the first episode, while *K. pneumoniae* was detected by blood culture. For case No. 176, the PCR-MCA assay verified the results of blood culture for the first infection episode but showed negative results for the second and third episodes because the pathogens identified by blood culture were not on the PCR-MCA list. For case No. 334, the result of the PCR-MCA assay was the same as that of blood culture for the first infection episode but detected one more pathogen than blood culture for the second infection episode.

**Table 4:** Results of blood culture and the PCR-MCA assay for the identification of multiple infections.

**Patient**	**Date**	**Time-to-positivity of blood culture (hours)**	**Blood culture**		**PCR-MCA**	
			**Pathogen 1**	**Pathogen 2**	**Pathogen 1**	**Pathogen 2**
**Simultaneous multiple infections during one blood culture investigation**						
3	2016/10/15	23.0	*E. faecalis*	*Citrobacter*	*E. faecalis*	*Citrobacter*
13	2016/10/15	21.3	*S. epidermidis*	*S. aureus*	*S. epidermidis*	*S. aureus*
43	2017/1/10	9.7	*E. coli*	*K. pneumoniae*	*E. coli*	*K. pneumoniae*
87	2017/1/20	10.5	*E. coli*	*K. pneumoniae*	*E. coli*	*K. pneumoniae*
105	2017/2/1	27.0	*K. pneumoniae*	*S. capitis*	*K. pneumoniae*	*S. capitis*
178	2017/4/25	10.3	*K. pneumoniae*	*S. pneumoniae*	*K. pneumoniae*	*S. pneumoniae*
327	2017/5/25	12.5	*E. coli*	*E. faecium*	*E. coli*	*E. faecium*
**Sequential multiple infections at different times during one hospitalization**						
9	2016/10/19	9.8	*K. pneumoniae*	–	–	–
	2016/11/2	10.8	*E. faecium*	–	*E. faecium*	–
	2016/11/7	13.5	*P. aeruginosa*	–	*P. aeruginosa*	–
10	2016/11/14	11.3	*E. coli*	–	*E. coli*	–
	2016/11/18	67.0	*C. albicans*	–	*C. albicans*	–
	2016/11/19	11.0	*A. baumannii*	–	*A. baumannii*	–
82	2016/12/6	11.9	*E. coli*	–	*E. coli*	–
	2016/12/8	24.7	*S. haemolyticus*	–	*S. haemolyticus*	–
91	2017/3/20	14.3	*K. pneumoniae*	–	*K. pneumoniae*	–
	2017/4/4	10.5	*E. coli*	–	*E. coli*	–
94	2017/5/15	12.5	*S. aureus*	–	*S. aureus*	–
	2017/5/20	24.3	*S. capitis*	–	*S. capitis*	–
133	2017/5/19	22.8	*E. faecium*	–	*E. faecium*	–
	2017/5/25	12.2	*A. baumannii*	–	*A. baumannii*	–
176	2017/5/26	69.6	*E. coli*	–	*E. coli*	–
	2017/5/27	70.2	*C. difficile*	–	–	–
	2017/5/30	34.6	*E. avium*	–	–	–
334	2017/5/31	36.4	*K. pneumoniae*	–	*K. pneumoniae*	–
	2017/6/14	12.0	*E. coli*	–	*E. coli*	*E. faecalis*

*PCR-MCA, real-time polymerase chain reaction coupled with multiplex probe melting curve analysis*.

### Clinical Characteristics of Patients With Discordant Results Between Blood Culture and the PCR-MCA Assay

Among the 2,844 episodes of suspected BSIs, there were eight episodes from eight cases for which the results of the PCR-MCA assay were inconsistent with those of blood culture. The clinical characteristics of these cases are shown in Table 5. Two cases (cases number 9 and 42) were identified with *K. pneumoniae* infection by blood culture, but the results of the PCR-MCA assay were negative. These two cases both came from surgical wards and had increased C-reactive protein levels; case No. 42 also had a decreased white blood cell counts. Six cases (cases number 121, 229, 314, 334, 354, and 355) were identified as having single infection by blood culture, but the results of the PCR-MCA assay showed multiplex DNAemia from two pathogens. These six cases both had increased procalcitonin levels, and cases number 354 and 355 also had increased white blood cell counts, while cases number 314, 334 and 355 had increased C-reactive protein levels. The time-to-positivity (TTP) of blood culture was 9.8 h in cases number 9 and 42, and 13.0, 25.0, 18.3, 12.0, 13.7, 6.5 h in cases number 121, 229, 314, 334, 354, and 355, respectively. These discordant results were further confirmed using a uniplex PCR assay and showed the same results as the PCR-MCA assay.

**Table 5:** Clinical characteristics of patients with discordant results between blood culture and the PCR-MCA assay.

**Patient**	**Blood culture**	**PCR-MCA**		**Uniplex PCR**		**Sex**	**Age**	**Principal diagnosis**	**WBC (10^**9**^/L)**	**CRP (mg/L)**	**PCT (ng/mL)**	**Time-to-positivity of blood culture (hours)**
	**Pathogen 1**	**Pathogen 1**	**Pathogen 2**	**Pathogen 1**	**Pathogen 2**							
9	*K. pneumoniae*	–	–	–	–	Female	88	Postoperative PTCD and insertion of bile duct stents by ERCP	9.6	18.78	0.23	9.8
42	*K. pneumoniae*	–	–	–	–	Female	58	Postoperative breast cancer	0.34	105.48	–	9.8
121	*E. cloacae*	*E. cloacae*	*A. baumannii*	*E. cloacae*	*A. baumannii*	Male	48	Acute non-ST-segment elevation myocardial infarction	6.78	–	1.49	13.0
229	*S. capitis*	*S. capitis*	*S. pneumoniae*	*S. capitis*	*S. pneumoniae*	Female	75	Chronic kidney disease	10.48	3.14	0.69	25.0
314	*E. cloacae*	*E. cloacae*	*A. baumannii*	*E. cloacae*	*A. baumannii*	Male	62	Bilateral severe carotid stenosis	9.13	61.81	4.18	18.3
334	*E. coli*	*E. coli*	*E. faecalis*	*E. coli*	*E. faecalis*	Female	71	Upper gastrointestinal hemorrhage	8.08	57.8	3.49	12.0
354	*P. aeruginosa*	*P. aeruginosa*	*E. coli*	*P. aeruginosa*	*E. coli*	Male	73	Acute cholangitis	13.62	–	73.79	13.7
355	*E. coli*	*E. coli*	*K. pneumoniae*	*E. coli*	*K. pneumoniae*	Male	73	Advanced ampullary cancer	18.21	206.78	>100.0	6.5

*PCR-MCA, real-time polymerase chain reaction coupled with multiplex probe melting curve analysis; WBC, white blood cell; CRP, C-reactive protein; PCT, procalcitonin; PTCD, percutaneous transhepatic cholangial drainage; ERCP, Endoscopic retrograde cholangiopancreatography*.

## Discussion

Delayed diagnosis of a pathogenic infection may increase the mortality rate of fatal sepsis. The earlier specific antibiotic administration is performed, the better the effect of the treatment and benefit to the patients (Dellinger et al., 2008). To date, the routine method for pathogen identification was blood culture, the whole procedure for which requires more than 2 days to perform and includes blood culture, Gram staining, plate culture and systematic microbial mass spectrometric identification. Because of the long duration of blood culture, empirical antibiotic therapy is administered by necessity (Singer et al., 2016), enhancing the problem of antibiotic resistance. In recent years, molecular biological methods have gradually been applied in pathogen detection in sepsis, reducing the detection duration while demonstrating excellent diagnostic performance (Rello et al., 2018).

The present study included 2,763 patients with 2,844 consecutive new episodes of suspected BSIs to evaluate a PCR-MCA assay for pathogen detection. The most common pathogen identified by blood culture was *E. coli*, followed by *K. pneumoniae, S. aureus, S. epidermidis, A. baumannii, P. aeruginosa, S. pneumoniae, C. albicans*, etc. The PCR-MCA assay could identify 11 kinds of gram-positive bacteria, 12 kinds of gram-negative bacteria, five kinds of fungi and two types of resistance genes. For the pathogens on the PCR-MCA list, the PCR-MCA assay discovered 99.2% (239/241) of the pathogens discovered by blood culture, with a specificity of 100% (2,575/2,575) and agreement of 99.9% (2,814/2,816) compared with the results of blood culture, suggesting that the assay presented excellent diagnostic performance compared with blood culture. For all the pathogens tested in clinical practice, the PCR-MCA assay also presented a good sensitivity of 88.8% (239/269) with a specificity of 100% (2,575/2,575) and agreement of 98.9% (2,814/2,844). For some prevalent pathogens, e.g., *E. coli, A. baumannii, P. aeruginosa, S. aureus, S. epidermidis*, and *S. pneumoniae*, the sensitivity of the PCR-MCA assay could reach 100%, while the specificity reached over 99%. Notably, for some samples with multiple pathogens identified simultaneously during one blood culture investigation, the PCR-MCA assay verified the results of blood culture, with an agreement rate of 100% for each. This finding may indicate that the PCR-MCA assay could be used to obtain early indication of multiple infections and help clinicians adopt the corresponding treatment strategy in a timely manner.

Discordance was observed in eight episodes of BSIs from eight cases. Two of them had negative results in the PCR-MCA assay but were identified with *K. pneumoniae* infection by blood culture. The TTP of both these cases was 9.8 h, much shorter than the average TTP of *K. pneumoniae* of 22 h (Ning et al., 2016). The TTP of an automated continuous blood culture system may indicate the original concentration of the organism (Da et al., 2016). It could be inferred that the original concentration of *K. pneumoniae* was sufficient, but the failure of the PCR-MCA assay to detect this organism may be due to the low sensitivity of this method compared to blood culture for *K. pneumoniae* detection. Samples of six other episodes were identified with one more pathogen than what was identified by blood culture. The TTP of blood culture of all these samples, which is defined as the length of time from the beginning of culture incubation to the detection of bacterial growth by an automated system (Tang et al., 2017), was ≤ 25 h. It could be inferred that the pathogen identified by blood culture was in dominance, which had a higher initial inoculum concentration or speed of growth, and its growth competitively inhibited others, known as “Jameson effect” (Mellefont et al., 2008). Non-dominant pathogens could also cause BSIs or originate from contamination.

The PCR-MCA assay is more advanced in some aspects than the existing blood culture techniques. First, <4 h were required to perform an entire PCR-MCA procedure. Pathogens of suspected BSIs could be identified within a short time, which will guide clinicians to adjust antibiotic therapy in a timely manner. In this way, the mortality of sepsis could be reduced, as well as the trends of resistance development. Second, the PCR-MCA assay could detect 28 kinds of pathogens simultaneously, which could prevent interference by the “Jameson effect” in mixed infection. In the blood culture process, identification and analysis of pathogens followed cultivation. When two or more pathogens are present in one sample, once the growth of dominant pathogens competitively inhibits others, the results of blood culture may falsely indicate single infection. Moreover, the PCR-MCA assay could detect two types of resistance genes, namely, *vanA* and *mecA*, along with pathogen detection, although none was identified in the present study. Third, the NPV of the PCR-MCA assay could reach 100% in detection of 27 kinds of pathogens on the panel except for *K. pneumoniae*. Even for pathogens not on the PCR-MCA list, the NPV reached over 99%. Since a sufficiently high NPV could provide high odds to exclude uninfected patients, unnecessary empirical antibiotic therapy could be avoided.

This study has several limitations that need to be addressed. First, not all of the pathogens that cause BSIs were included in this assay, and only 88.8% of the prevalent pathogens were identified, limited by the panel of detectable germs. Additional kinds of prevalent pathogens should be incorporated into this assay before clinical application. Second, the number of targeted pathogens, i.e., *Serratia marcescens, Proteus mirabilis, Streptococcus pyogenes, Listeria monocytogenes, Candida parapsilosis, Candida krusei*, and *Aspergillus fumigatus*, was insufficient for evaluation of diagnostic performance. The detection rate of the resistance genes *mecA* was 0, relatively low compared with the data reported by the China Antimicrobial Surveillance Network (http://www.chinets.com/), in which the prevalence of methicillin-resistant *Staphylococcus aureus* (MRSA) could be as high as 45.8% and the detection rate of *mecA* in MRSA could be 93.6% (Chen et al., 2012). That may be explained by the small number of MRSA (*n* = 4) in our study site, as well as the probability of a *mecA*-negative strain of *Staphylococcus* species with high-level beta-lactam resistance which has been reported (Banerjee et al., 2010). In addition, no vancomycin-resistant *Enterococcus* (VRE) species was identified in phenotypic susceptibility tests, consistent with the PCR-MCA result showing that the *vanA* gene was not detected. According to the China Antimicrobial Surveillance Network, the prevalence of VRE was 2.3%, and the detection rate of *vanA* in VRE could be 73.9% (Zhao et al., 2012). Larger samples are needed to verify the efficiency and accuracy of the PCR-MCA assay in future studies. Third, the resistance genes *vanA* and *mecA* included in the PCR-MCA assay were related to only gram-positive bacteria, so additional kinds of prevalent resistance genes should be incorporated into this assay for further study. Finally, samples with discordant results were further verified using a uniplex PCR assay and showed the same results as the PCR-MCA assay. Since the uniplex PCR detection system was built following the same principle as the PCR-MCA assay, the confirmation results may not be sufficiently objective, and a third detection method is necessary for evaluation. In addition, several positive PCR-MCA results but negative blood culture results were observed in this study, though the agreement rates of these two assays were high in general. Cross-reaction with other potential microorganisms in this assay should be investigated more extensively to promote clinical application.

In conclusion, our study demonstrated that the PCR-MCA assay is valuable proof of principle, simultaneously identifying 28 kinds of pathogens and two resistance genes in patients with suspected BSIs within a few hours. This novel assay exhibited considerable sensitivity and specificity and covered 88.8% of the pathogens in clinical practice. Rapid identification of pathogens involved in BSIs could help clinicians to make the correct diagnosis and guide antibiotic administration. Furthermore, additional kinds of prevalent pathogens could be selected to incorporate into this assay, which could be applied in clinical practice someday.

## Data Availability Statement

All datasets generated for this study are included in the manuscript/supplementary files.

## Ethics Statement

This study was approved by the Institutional Ethics Committee of Zhongshan Hospital, School of Medicine, Xiamen University, and was in compliance with national legislation and the Declaration of Helsinki guidelines. Written informed consent was obtained from the patient or, from next of kin or an independent patient advocate if the patient lacked this capacity.

## Author Contributions

YX and XS wrote the main manuscript text. Q-FZ and Y-HY contributed to samples collection and laboratory tests. T-CY performed the statistical analysis. J-JN conceived the study and revised the manuscript.

### Conflict of Interest

The authors declare that the research was conducted in the absence of any commercial or financial relationships that could be construed as a potential conflict of interest.
